# Novel phonocardiography system for heartbeat detection from various locations

**DOI:** 10.1038/s41598-023-41102-8

**Published:** 2023-09-01

**Authors:** Rene Jaros, Jiri Koutny, Martina Ladrova, Radek Martinek

**Affiliations:** Department of Cybernetics and Biomedical Engineering, Faculty of Electrical Engineering and Computer Science, VSB–Technical University of Ostrava, 17. listopadu, 708 00 Ostrava, Czechia

**Keywords:** Data processing, Biomedical engineering

## Abstract

The paper presents evaluation of the proposed phonocardiography (PCG) measurement system designed primarily for heartbeat detection to estimate heart rate (HR). Typically, HR estimation is performed using electrocardiography (ECG) or pulse wave as one of the fundamental diagnostic methodologies for assessing cardiac function. The system includes novel both sensory part and data processing procedure, which is based on signal preprocessing using Wavelet Transform (WT) and Shannon energy computation and heart sounds classification using K-means. Due to the lack of standardization in the placement of PCG sensors, the study focuses on evaluating the signal quality obtained from 7 different sensor locations on the subject’s chest and investigates which locations are most suitable for recording heart sounds. The suitability of sensor localization was examined in 27 subjects by detecting the first two heart sounds (S1, S2). The HR detection sensitivity related to reference ECG from all sensor positions reached values over 88.9 and 77.4% in detection of S1 and S2, respectively. The placement in the middle of sternum showed the higher signal quality with median of the proper S1 and S2 detection sensitivity of 98.5 and 97.5%, respectively.

## Introduction

Heart activity monitoring using non-electrical principles is becoming more and more popular due to many advantages in applications such as telemedicine, whole-day monitoring, easy-to-use sensors or measuring in specific environments. To follow these up-to-date requirements, many studies have started to deal with development and implementation of various sensors into unobtrusive monitoring systems. One of extensive developed areas during recent years is measuring using phonocardiography (PCG), i.e., record of heart sounds (HS) and murmurs that arise due to a resonance of heart structures and blood^1, 2^. The four heart sounds are distinguished (S1, S2, S3, and S4), whereas the most prominent heart sounds are caused by closing of heart valves.

The heart sounds spread through the whole chest cavity and it is possible to record them using a microphone, which makes a heartbeat detection low-cost and unobtrusive for patient, since there is no need for skin preparation and applying multiple disposable electrodes as when using ECG. The classification and interpretation of features extracted from the PCG signal is the subject of scientific research conducted by many authors^1–4^. However, the sensor location for PCG recording is not standardized and the exact placement of sensor is usually not described in the studies^5–7^. Localization of twelve cardio-microphones was investigated more in details only by Fontecave-Jallon et al.^7^ on two subjects.

The proposed study aims to compare the quality of signals measured from different positions within the chest and find the optimal one for PCG recording using the novel measurement system. The study builds upon the previous research^8^ focusing on using PCG as an alternative method for triggering sequences of magnetic resonance imaging (MRI), which is a widely researched area due to insufficient heartbeat detection by standardly used ECG (with gradually increasing magnetic field strength utilized in MRI, ECG is more devaluated by artifacts^9^). The previous study confirmed a suitability of the proposed sensor for monitoring in MRI, however, there were limitations in placement of the sensor due to highly individual body shape of subjects. Therefore, the primary objective of the present study is to gain insights into the appropriate placement of the proposed sensor for future utilization of PCG in MRI triggering, but also in other applications. These detected heart sounds were used for further calculation of heart rate (HR) and compared to reference values obtained from the standard ECG system.

The paper is divided into five sections. Section State of the Art presents methods for PCG preprocessing with the focus on wavelet transform and signal segmentation. Section Material and Methods describes the used measurement system, placement of microphones, experimental setup, evaluation parameters and methods used for signal preprocessing and HS detection. Section Results presents the obtained results, which are discussed in Section Discussion, together with shortcomings of the proposed method and motivation for further research.

## State of the art

The PCG signal, like other biological signals, can be evaluated in time, frequency or time-frequency domain^10^. The signal is usually preprocessed using some of the filtration methods and divided into four parts (S1, systole, S2, diastole) using segmentation that is a frequent subject of research^2, 11^. The PCG processing and analysis can include different advanced methods, such as Mel-Frequency Cepstral Coefficients (MFCCs), Hilbert Vibration Decomposition (HVD), Short-Time Fourier Transform (STFT), Empirical Mode Decomposition (EMD), Machine Learning, or other intelligent systems^12^. Based on the literature, the PCG signal was preprocessed in this study using linear filters and wavelet transform thanks to their verified results and wide accessibility^13–15^. The following subsections present the state-of-the-art of the chosen processing methods more in detail.

### Signal filtration

The PCG signal is a non-stationary biological signal, containing noise and many types of artifacts arising from external (environment, sensor movement) or internal (breathing, chest movement, coughing, sound manifestation of intestines or stomach) effects^12^. These artifacts can effect the resulting signal, thus the proper extraction of the desired information is needed. For elimination of low-frequency and high-frequency interference, linear filters can be used, such as Butterworth or Chebyshev^5, 16, 17^. Cut-off frequency of about 20 Hz is usually used for high-pass filter, while cut-off frequencies for low-pass filtration are set depending of the purpose of analysis; 100 Hz for S1/S2 detection and up to 800 Hz for evaluation of heart murmurs^18^.

### Wavelet transform

Discrete Wavelet Transform (DWT) is a widely used tool for preprocessing of biological signals. The signal is decomposed using the adequate type of maternal wavelet into detailed and approximate coefficients (wavelet decomposition). The used type of maternal wavelet and level of decomposition differ across studies. Usually, the wavelets Daubechies (db), Discrete Meyer, Coiflet (coif), Morlet, or Symlet (sym) are used. Some studies also verified the performance of the wavelets Harr, Biorthogonal (bior), or Reverse Biorthogonal (rbio), see Table 1. Some of the studies compare the different wavelet types from the perspective of performance^12, 15, 19–21^. Therefore, Table 1 includes all tested wavelets together with the ones with the best results obtained. One can see that the wavelets most often reaching the best results were from Daubechies family (db4/db6) with the decomposition level from 4 do 7.

### Signal segmentation

The signal segmentation and associated localization of S1 and S2 are important steps for the PCG signal analysis. The segmentation methods can be characterized as:

- *Indirect*—PCG is measured together with ECG (or pulse wave) reference, when stamps from ECG signal (moment of occurrence of R and T waves) are used to identify the individual heart cycles and the corresponding heart sounds^2, 11, 12, 22, 23^,
- *Direct*—only PCG is measured, which needs to be transformed into the domain that allows increasing of S1 and S2 amplitude^2^.

**Figure 1:**
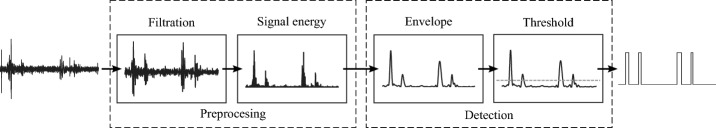
General scheme of direct PCG segmentation^2^.

The direct segmentation is more complex, see Fig. 1. The segmentation methods in time domain provide direct and the most reliable localization of heart sounds, however, they demand high-quality signal preprocessing due to their high susceptibility to noise^10^. From that reason, using the time-frequency domain is quite popular^2, 23, 24^, where the increased spectral power on frequencies from 40 Hz to 100 Hz is visible. This approach includes S1 and S2 detection based on signal envelope, which was dealt in^5, 7, 14, 16^.

**Table 1:** Frequently used wavelets for PCG processing..

Study	Year	Used wavelet (best result)	Tested wavelets
Chowdhury et al.^19^	2019	db18 (lvl 6)	db1–db20, coif1–coif5, bior+rbio (15 wavelets)
Nabih Ali et al.^4^	2017	db10, discrete Meyer	db1–db10, Harr, sym2–sym10,
		(lvl 4)	coif1–coif5, bior, rbio, discrete Meyer
Kumar Jain et al.^25^	2017	coif5 (lvl 4, 5)	–
Kumar Jain et al.^26^	2016	coif5 (lvl 4, 5)	–
Pedrosa et al.^27^	2014	Morlet	–
Marques et al.^20^	2013	db6, db9 (lvl 10)	db1–db40
Deng et al.^28^	2012	db6 (lvl 4)	–
Song et al.^29^	2012	db6 (lvl 4)	–
Kouras et al.^30^	2012	db10 (lvl 6)	–
Meziani et al.^13^	2012	db7, Morlet	–
Tu et al.^31^	2010	db6 (lvl 7)	–
Babaei et al.^21^	2009	db4 (lvl 4)	Haar, db4, coif3, sym5, rbio 1.5 (lvl 1–10)
Quiceno et al.^32^	2008	coif4 (lvl 8), db6 (lvl 6)	–
Kumar et al.^33^	2006	db6	–
Wang et al.^34^	2005	db4 (lvl 7)	–
Messer et al.^15^	2001	coif4, coif5, db11, db14, db20,	db1–db45, sym1–sym15,
		sym9, sym11, sym14 (lvl 5)	coif1–coif5
Liang et al.^35^	1998	db6 (lvl 5)	–
Huiying et al.^14^	1997	db6 (lvl 5)	–

The direct segmentation is easy to perform in healthy subjects^36^, assuming that systole is shorter than diastole. However, systole and diastole duration is dependent on heart rate^23^, so the detection can be problematic in patients with tachycardia^2^. In some cases, it can be difficult to ensure elimination of extra peaks and preserving of true heart sounds, which is crucial for correct direct segmentation^37^.

## Material and methods

### Measurement system

**Figure 2:**
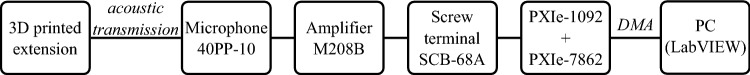
Block diagram of the DAQ system.

The used data acquisition (DAQ) system (see Fig. 2) includes chassis PXIe-1092^38^ with a Field-Programmable Gate Array (FPGA) module from National Instruments (NI)–NI PXIe-7862^39^, which allows to measure 16 analogue input signals with 16bit resolution and maximum sampling frequency of 1 MS/s per channel. This module was connected to analogue inputs via terminal block SCB-68A^40^. For data transmission between PC and FPGA module, the direct memory access (DMA) was used.

Seven microphones *GRAS 40PP-10* with a frequency range from 10 Hz to 20 kHz and sensitivity of 50 mV/Pa^41^ were used to sense and convert the acoustic signal. A relative connection of microphones was used, which allows the sensing of pressure changes of the chest movement at the location of sensor^5, 6^. In order to supply the microphones with the required current of 2 to 20 mA, they were connected to the terminal block via an eight-channel IEPE amplifier M208B, the gain of which was set to 0 dB (gain = 1) and was used only for power supply.

**Figure 3:**
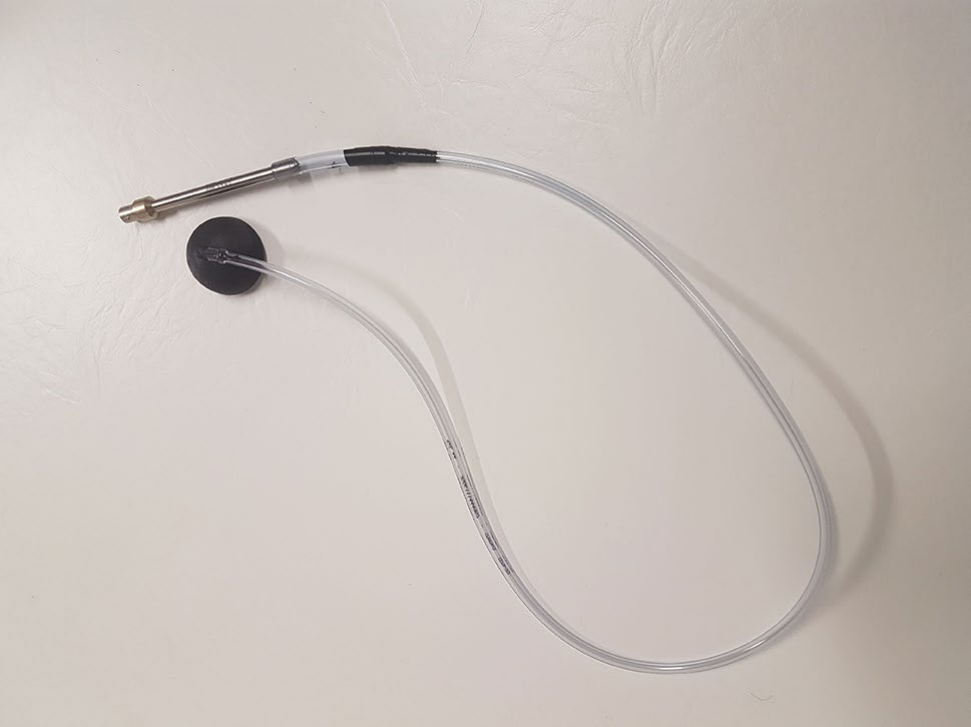
3D printed extension connected to the microphone.

The conduction to the microphone is made through the air-filled tube. An air chamber for conduction of pressure changes to the microphone was created by the 3D-printed sensor of the hemisphere shape with a diameter of 40 mm and PVC tube connecting the sensor with microphone, see Fig. 3. The frequency analysis of the measured signal showed that the proposed system (sensor with a diameter of 40 mm and tube length of 1 m) absorbs signals with frequency higher than 100 Hz. Because the heart sounds S1 and S2 are composed of frequencies lower than 100 Hz, the system is suitable for the HR detection.

The whole sensory part of system that is in contact with patient is made of low-cost and non-electrical materials, which can be advantageous for many applications, such as use within magnetic resonance (MR) environment, when the use of electrical conductive parts could cause an injury of patient^8^.

### Sensors placement

**Figure 4:**
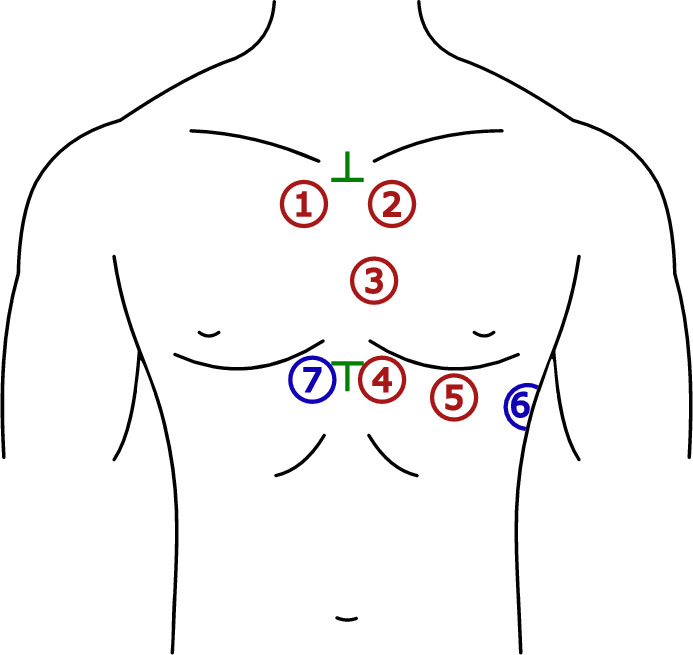
The selected auscultation points for PCG recording (P1–P7): red—traditional auscultation areas, blue—additional areas, green—0reference points.

Many studies focusing on detection of HS do not specify the exact sensor placement^17, 42–48^. The placement of sensors is not standardized, but certain auscultation points provide the best audible heart sounds. Suitable sensor placement is researched in^7, 49–51^. Seven sensors (labeled as P1-P7) were used in the present study for multichannel PCG measurement, see Fig. 4. Sensors were positioned near the heart valves (P1, P2, and P3), and further toward the apex of the heart (P4, P5). Positions P6 and P7 are not directly over the heart, serving to verify if a quality PCG signal can be obtained even in those locations. Positions P1-P5 were chosen to reflect traditional auscultation areas^49, 52^:

- P1—manubrium, parasternally right (“aortic area”),
- P2—manubrium, parasternally left (“pulmonary area”),
- P3—middle of the sternum, parasternally left (“Erb’s auscultation point”),
- P4–P6—xiphoid process, parasternally left (“tricuspidal area”),
- P7—xiphoid process, parasternally right.

Cranial and caudal endings of the sternum were chosen as reference points for a placement of sensors (see green marks in Fig. 4).

### Dataset

The measurement was preformed on 27 volunteers (10 women and 17 men) aged from 21 to 29 years. All collected information about subjects, such as age, height, weight, and body mass index (BMI) are summarized in Table 2. One of the probands (#22) suffers from heart murmurs of the intensity of 1/6 with a high blood pressure and tachycardia. All of the remaining signals were without pathology.

**Table 2:** General information about the tested subjects..

Parameter	Range (median)
Age (years)	21–29 (24)
Height (cm)	1.58–1.94 (1.73)
Weight (kg)	50–99 (69)
BMI (kg m$^{-2}$)	18.2–28.1 (23.4)

All experiments were performed in accordance with the relevant regulations and approved by the Ethics Committee of the Technical University of Ostrava. Experiments were completely safe, using certified HW from National Instruments. An informed consent form was prepared for this study, which all participants signed to confirm that they agreed to the publication of results, where all their data will be anonymised. All data generated or analysed during this study are included in this published article (and its supplementary information files).

The study is limited to a small sample of young adult participants, since the aim of this study is to test the usability of the proposed sensors and determine the most suitable position for measuring the PCG signal. It is important to note that having only one pathological sample is not sufficiently indicative, and therefore, a larger sample of participants that encompasses various age groups and includes a wider range of pathological signals will be a goal of future studies to obtain more meaningful results (see Discussion section).

Each volunteer underwent 10-min measurement in the supine position using the proposed measurement system. The sensors were located according to Fig. 4 and secured by three stretch belts. For measuring reference ECG, a clip-on or floating electrodes were used. All the signals were sampled with a frequency of 2.56 kHz. Parameters of the measured signals are summarized in Table 3 and all signal processing steps were realized using LabVIEW software, National Instruments.

**Table 3:** Parameters of measured signals.

Parameter	Value
Measurement time	approx. 10 min
Sampling rate	2.56 kHz
Voltage range	± 10 V
DAQ system resolution	16bit (0.3 mV)
Microphone resolution	50 mV/Pa
# of channels	8 (ECG + 7 PCG)

### PCG signal preprocessing

For preprocessing, PCG signal was filtered to highlight the desired heart sounds using band-pass filter (BPF) and DWT. Then, calculation of Shannon energy of the signal and its envelope was used for HS detection, see Fig. 1.

#### Band-pass filter

**Figure 5:**
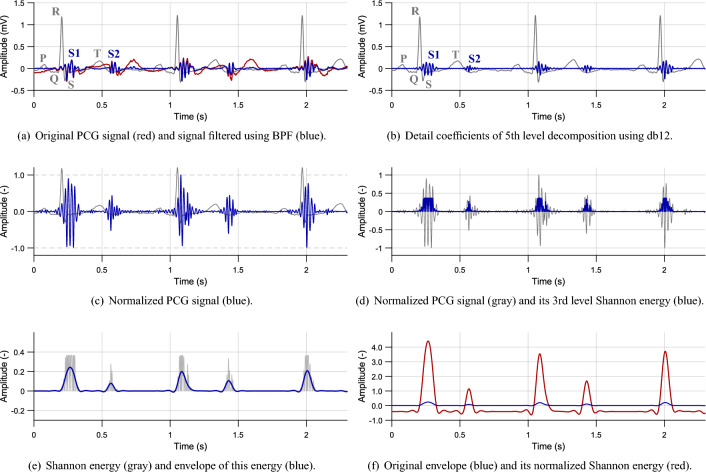
PCG preprocessing steps (volunteer #2, position P3).

First, the signal was filtered using Butterworth BPF of the 2nd order with a lower cut-off frequency of 2 Hz and higher cut-off frequency of 100 Hz, see Fig. 5a. Elimination of low frequencies is performed primarily to suppress motion artifacts and compensate isoline of the signal. Frequencies higher than 100 Hz contain undesired noise.

#### Discrete wavelet transform

As the next step, the signal was processed using DWT. Preliminary experimental measurements were first performed to find out a suitability of different maternal wavelets for the obtained PCG signals. In accordance with a literature (see Table 1), the signal was filtered using available wavelet functions (*Advanced Signal Processing Toolkit* by NI LabVIEW) from the families of Daubechies, Haar, Biorthogonal, Coiflet, and Symlet. Based on these experimental measurements, Daubechies (db12) wavelet with a decomposition level of 5 was chosen for the final filtering process due to the best results compared to other tested wavelets. The example result of WT filtering is shown in Fig. 5b.

#### Normalization

The filtered signal was normalized to values from $-1$ to 1 according to Eq. (1):

1 $$\begin{aligned} x_{n}(i)=\frac{x(i)}{\max (|x(i)|)}, \end{aligned}$$

where $x_{n}(i)$ is normalized signal and *x*(*i*) is the original one, see Fig. 5c^16, 36^. The normalization was performed within 1 s sections, since its applying on the whole signal can be inaccurate due to presence of undesired high amplitudes, e.g., caused by movement.

#### Signal energy

From the normalized signal, Shannon energy of the 3rd order was calculated according to Eq. (2):

2 $$\begin{aligned} E_{S}(i)=-|x_{n}^{3}(i)| \cdot \log |x_{n}^{3}(i)|, \end{aligned}$$

where $E_{S}(i)$ is the output energy and $x_{n}(i)$ is the normalized signal^16^. Shannon energy highlights mean values of the signal and suppresses low values together with values close to 1, see Fig. 5d. The benefit of Shannon energy compared to absolute value is the increasing of differences in sounds with middle and high intensity, which simplifies the further localization of heart sounds^5, 16, 36, 42^.

#### Signal envelope

The envelope was created by filtration of signal energy using Butterworth low-pass filter (LPF) of the 2nd order with zero phase shift and cut-off frequency of 10 Hz, see Fig. 5e. To increase an envelope amplitude, the normalized Shannon energy (see Fig. 5f) was determined using Eq. (3):

3 $$\begin{aligned} E_{N}(t)=\frac{E_{S}(t)-\mu }{\sigma }, \end{aligned}$$

where $E_N$ is the resulting energy, $\mu$ is mean value of the signal $E_S$ and $\sigma$ is standard deviation of the signal $E_S$.

### Heart sound detection

The peaks from this preprocessed signal were detected and further segmentation was applied, as shown in Fig. 1. Further, the intervals between the detected peaks were classified as systole or diastole. The peak before systole was assigned as S1 and the one before diastole as S2.

#### Peak detection

**Figure 6:**
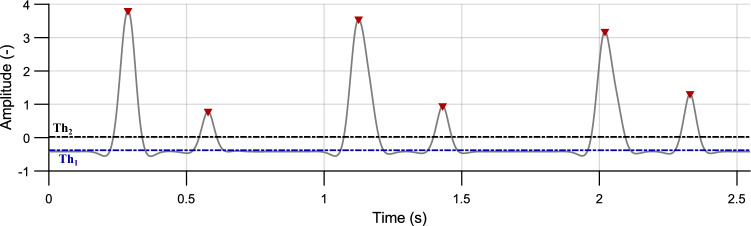
Envelope of the PCG signal (gray curve) with detected peaks (red) above the $Th_{1}$ threshold (blue dashed line). The $Th_{2}$ threshold (black dashed line) is intended to further peaks classification (volunteer #2, position P3).

First, peaks of the envelope located above the defined threshold $Th_{1}$ were detected (see Fig. 6):

4 $$\begin{aligned} Th_{1}=-0.9\cdot \frac{\mu }{\sigma }, \end{aligned}$$

where $\mu$ is a mean value and $\sigma$ is a standard deviation of the signal $E_{S}$. The value of this threshold is 90 % of the signal shift (see Eq. (3)). This threshold allows for the detection of weak heart sounds as well as noise. Peaks detected using this threshold are not primarily labelled as heart sounds but are utilized for further completion based on the criteria described below. Duration of the heart sounds is usually about 150 ms. If the interval between two consecutive peaks was shorter than 150 ms, the peak with lower amplitude was removed. With this process, even low-amplitude false peaks were detected and were confirmed by the further processing.

#### Peak classification

The next step required to assign the detected peaks to the individual heart sounds (S1 or S2) using K-means cluster analysis and classify the false heart sounds. During the rest HR (lower than 130 bpm), the interval S1–S2 (systole) is shorter than the following interval S2–S1 (diastole)^23^. The intervals between peaks with higher amplitude than the threshold $Th_{2}$ (see Fig. 6) were investigated:

5 $$\begin{aligned} Th_{2}=\overline{E_{S}}, \end{aligned}$$

where $\overline{E_{S}}$ is a mean value of the signal $E_{S}$.

**Figure 7:**
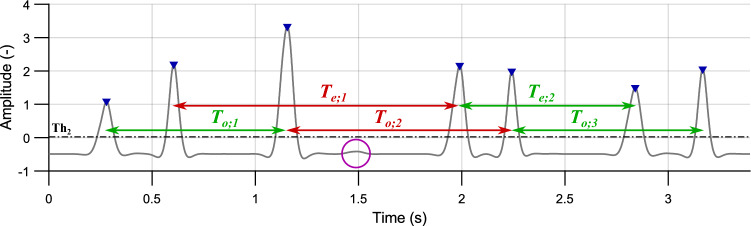
Intervals between even ($T_{e;n}$) and odd ($T_{o;n}$) peaks. Correctly detected intervals (which correspond to the HR) are marked in green. Intervals marked in red are detected incorrectly due to non-detected peak (marked with a purple circle); volunteer #4, position P3.

Under ideal conditions, the intervals S1–S1 (or S2–S2) take place between even (or odd) peaks, representing heart rate. However, there occurred some extra peaks and some non-detected peaks, see Fig. 7. Therefore, the parameter *presumed heart period* calculated as modus $\widehat{T}_{S-S}$ of the intervals between even and odd peaks was defined:

6 $$\begin{aligned} \widehat{T}_{S-S}=Mod\bigg (\Big [\left[ T_{o; 1}, \ldots , T_{o; n}\right] \left[ T_{e; 1}, \ldots , T_{e; n}\right] \Big ]\bigg ), \end{aligned}$$

where $T_{o; n}$ and $T_{e; n}$ are intervals between two odd and even peaks, respectively. This value was used for approximate determination of heart rate and selection of primary centroids for classifying the intervals using K-means method.

For K-means cluster analysis, the number of clusters was selected as ${k=3}$. Intervals are divided into clusters, which can be called “systole”, “diastole”, and “longer intervals”. The primary centroids *C* were selected as ${C_{1}=0,35\cdot \widehat{T}_{S-S}}$, ${C_{2}=0,65\cdot \widehat{T}_{S-S}}$, and ${C_{3}=\widehat{T}_{S-S}}$ for systole, diastole, and longer intervals, respectively. Values $C_{1}$ and $C_{2}$ represent 35% and 65% of $\widehat{T}_{S-S}$, which is approximate ratio of duration of systole and diastole during rest heart rate^23^.

**Figure 8:**
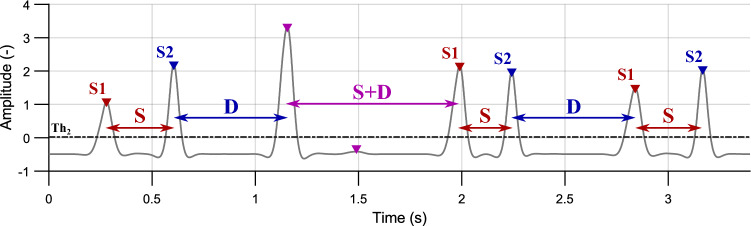
Interval-based peak assignment. S1 before systole (S, red), S2 before diastole (D, blue) and non-classified peaks before longer intervals and below the threshold $Th_{2}$ (purple); volunteer #4, position P3.

Peaks located before the interval “systole” are marked as S1. Similarly, the peaks before the interval “diastole” are marked as S2. The peaks located before “longer intervals” or under the threshold $Th_{2}$ stayed not classified in this step, see Fig. 8.

#### Peak confirmation

**Figure 9:**
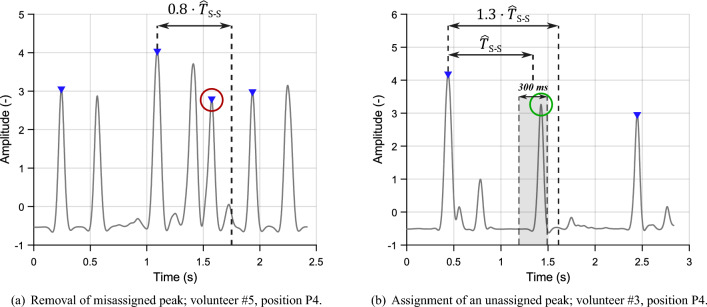
Conditions for peak removal and assignment.

Based on two conditions according to^53^, the false peaks were removed and non-detected peaks were assigned, see Fig. 9. Both conditions count with the *presumed heart period* $\widehat{T}_{S-S}$:

- *Removal of false peaks*—if the interval between consecutive peaks S1–S1 (or S2–S2) is shorter than ${0.8\cdot \widehat{T}_{S-S}}$, the second peak is marked as “non-classified peak”. This situation is shown in Fig. 9a.
- *Classification of non-classified peaks*—when the interval between S1–S1 (or S2–S2) is longer than ${1.3\cdot \widehat{T}_{S-S}}$, as shown in Fig. 9b, the interval is checked whether there is the missed peak.

Around the position, where the peak should occur (position of the assigned peak $+\;\widehat{T}_{S-S}$), window of the length of 300 ms (150 ms in each direction) was located. If there was found a non-classified peak, it was assigned to the same category as the previous one. If there were more peaks than one within the window, the one closer to the center of the window was added.

### Processing of reference ECG

The reference ECG was filtered using two Butterworth filters of the 2nd order with zero shift:

- *Band-pass filter* was used for elimination of artifacts caused by motion and respiratory activity with cut-off frequencies of 1 and 150 Hz.
- *Band-stop filter* was used to remove power line interference (50 Hz) with cut-off frequencies of 48 and 52 Hz.

The R peaks were detected with using adaptive threshold, which varied for the individual windows of 5 s, when the threshold was set as 70% of the maximum amplitude within the window. Peaks falsely marked as R peak and non-detected peaks were removed or added, respectively, according to the similar conditions like in the case of removal and addition of heart sounds^53^.

After the automatic detection of R peaks, their positions were thoroughly verified by the authors to avoid any incorrect assessments. The chosen detection method demonstrated accurate performance in the examined sample of healthy subjects. For pathological signals, more precise algorithms would be necessary, such as^54–56^.

### Evaluation parameters

This section describes the parameters used for evaluation of HS detection performance on the individual locations (P1–P7). Also, there are described the methods used for comparison of heart rate values obtained from PCG with the ones from ECG.

#### Peak detection

**Figure 10:**
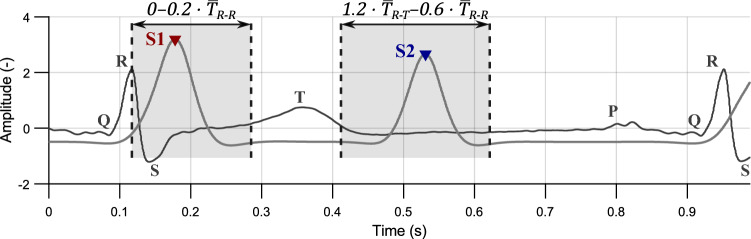
Intervals for evaluation of HS detection quality; volunteer #4, position P3.

For evaluation of HS detection performance, reference ECG was used. Based on^23^, where the indirect PCG segmentation is described in detail, time windows of the expected occurrence of heart sounds were selected, see Fig. 10. Time duration of these windows depends on heart rate (i.e., it shortens with increasing HR).

The S1 should be located in the interval of 0–${0.2\cdot \overline{T}_{R-R}}$ after the detected R peak, where $\overline{T}_{R-R}$ is a median of intervals between two consecutive R peaks. Duration of this interval is around 160 ms (median of all measurements is 168 ms).

For S2 detection, the interval was set from ${1.2\cdot \overline{T}_{R-T}}$ (end of T wave) to ${0.6\cdot \overline{T}_{R-R}}$, where $\overline{T}_{R-T}$ is a median of intervals between R peak and T wave maximum. Duration of this interval was longer than for S1 detection—up to 200 ms (median of all measurements is 189 ms).

Based on the success of peak detection within the selected window, parameters such as True Positive (TP), False Positive (FP), and False Negative (FN) values were calculated:

- TP indicates the number of detected peaks within the window,
- FP indicates the number of detected peaks out of the window,
- FN indicates the number of non-detected peaks within the expected interval.

These parameters were then used for calculation of sensitivity (SE) according to Eq. (7), which is one of the most significant statistical parameter stating the probability of the occurrence of the heart sound in the selected interval^2, 10^. According to Eq. (8), Positive Predictive Value (PPV) is determined, i.e., a probability of the correct peak detection.

7 $$\begin{aligned} SE=\frac{TP}{TP+FN}\cdot 100\;(\%). \end{aligned}$$

8 $$\begin{aligned} PPV=\frac{TP}{TP+FP}\cdot 100\;(\%). \end{aligned}$$

#### Heart rate evaluation

The signal from the position with the best values of SE and PPV was used to determine heart rate $H\!R_{S1}$ (or $H\!R_{S2}$) from the intervals S1–S1 (or S2–S2). Heart rate obtained from R peaks of ECG $H\!R_R$ was used as a reference. Individual $H\!R$ values were then calculated as follows:

9 $$\begin{aligned} H\!R_{S1}=\frac{60}{\overline{T}_{S1-S1}}\;(BPM), \end{aligned}$$

where $\overline{T}_{S1-S1}$ is median of intervals between two consecutive S1 peaks in the window of 2 s and $H\!R_{S1}$ is heart rate with unit *BPM* (beats per minute, $min^{-1}$). Similarly, $H\!R_{S2}$ and $H\!R_{R}$ can be determined using periods $T_{S2-S2;n}$ (S2–S2 interval) and $T_{R-R;n}$ (R–R interval).

The calculated values of $H\!R_{S1}$ and $H\!R_{S2}$ were then compared to reference $H\!R_{R}$ using pair Wilcoxon test, since the normality of data was rejected in some cases (Shapiro-Wilk test, *p*-value $=$ 0.05)^57^. The *p*-values less than 0.05 were considered to indicate a statistically significant difference.

## Results

**Figure 11:**
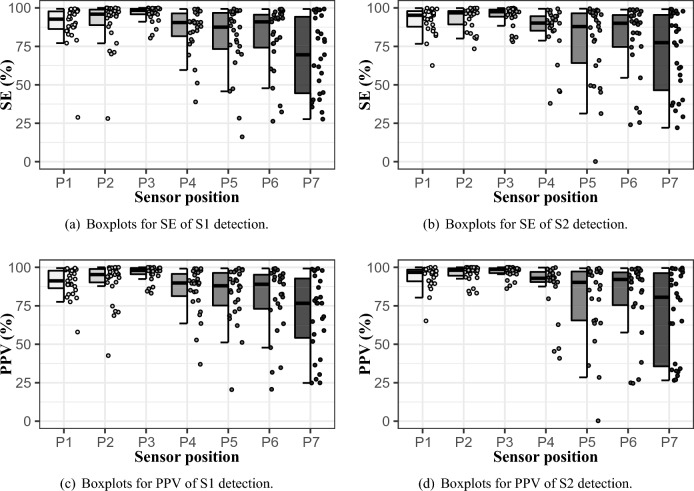
SE and PPV of HS detection in hybrid boxplots.

Quality of HS detection in the individual locations (P1–P7) was evaluated using SE and PPV. Their results for all measured subjects are presented by range of their minimum and maximum (min;max), median, and interquartile range (*IQR*), defined as:

10 $$\begin{aligned} IQR=Q_3-Q_1, \end{aligned}$$

where $Q_1$ is the first quartile and $Q_3$ is the third quartile of the SE and PPV values. All these parameters are summarized in Table 4 and represented by hybrid boxplots in Fig. 11a,c for S1 detection and in Table 5 and Fig. 11b,d for S2 detection.

**Table 4:** SE and PPV parameters for S1 detection.

Sensor position	SE (%)		PPV (%)	
	(min; max)	Median (IQR)	(min; max)	Median (IQR)
P1	(28.8; 99.4)	**92.7** (85.8; 97.8)	(57.9; 99.5)	**91.2** (85.9; 97.8)
P2	(28.1; 100)	**95.9** (88.3; 99.2)	(42.6; 100)	**95.3** (90.2; 99.0)
P3	(80.4; 100)	**98.5** (95.8; 99.5)	(83.2; 100)	**98.0** (95.5; 99.6)
P4	(38.9; 99.3)	**90.5** (80.5; 96.8)	(37.0; 99.2)	**89.9** (79.9; 96.1)
P5	(16.2; 99.4)	**87.5** (72.4; 97.0)	(20.5; 99.4)	**88.0** (74.1; 96.7)
P6	(26.3; 99.2)	**90.9** (72.9; 95.7)	(20.8; 99.3)	**89.0** (72.6; 95.6)
P7	(27.7; 99.3)	**69.6** (44.3; 94.6)	(24.8; 99.2)	**76.7** (53.0; 93.6)

Significant are in value [bold].

**Table 5:** SE and PPV parameters for S2 detection.

Sensor position	SE (%)		PPV (%)	
	(min; max)	Median (IQR)	(min; max)	Median (IQR)
P1	(62.5; 99.9)	**95.3** (87.2; 97.9)	(65.2; 100)	**96.7** (90.8; 98.6)
P2	(73.4; 99.9)	**96.8** (89.0; 98.1)	(82.9; 100)	**98.1** (94.1; 99.4)
P3	(78.0; 99.9)	**97.5** (94.3; 99.1)	(86.2; 100)	**98.7** (96.3; 99.5)
P4	(37.9; 99.4)	**90.1** (85.0; 94.6)	(40.9; 99.9)	**92.9** (90.4; 97.5)
P5	(0.1; 99.5)	**87.9** (63.3; 96.7)	(0.2; 99.4)	**90.2** (65.3; 97.3)
P6	(24.0; 98.9)	**90.0** (74.5; 95.5)	(24.6; 99.4)	**92.1** (74.9; 97.0)
P7	(22.1; 99.1)	**77.4** (43.3; 95.9)	(26.5; 99.6)	**80.5** (34.9; 96.5)

Significant are in value [bold].

### HR detection quality

**Figure 12:**
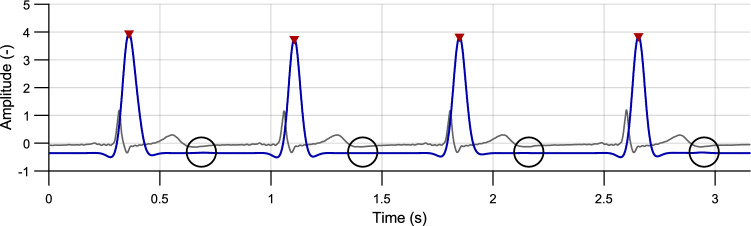
Faulty detection due to low S2 amplitude—reference ECG (gray curve), PCG envelope (blue curve) with detected peaks (red) and expected positions of S2 (black circles); volunteer #25, position P5.

The best performance of HS detection was reached on position P3, both in S1 and S2, as can be seen in Tables 4, 5 and Fig. 11. Contrary, the worst detection quality was obtained from position P7 in both cases. Low values of SE and PPV in positions P1 and P2 are present sporadically, so these locations can be also considered suitable. More often false detection occurred on positions P4, P5, and P6. In one extreme case (subject #25), SE and PPV values of S2 detection approached zero. The S2 was less accurately detected also in subject #22, who suffers from heart disease. The both described cases arised due to decreased S2 amplitude during signal normalization, see Fig. 12.

### Statistical comparison of HR

**Figure 13:**
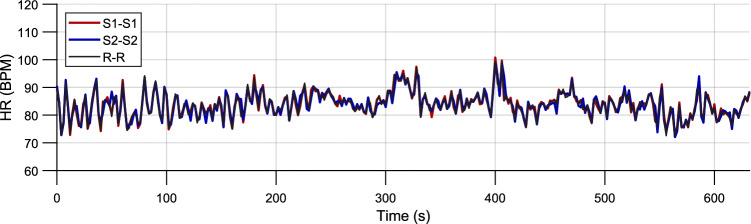
Heart rate calculated from the intervals between S1 peaks (red), S2 peaks (blue) and R peaks (black).; volunteer #26, position P3.

The $H\!R$ values (see Fig. 13) obtained by PCG ($H\!R_{S1}$, $H\!R_{S2}$) from position P3 (with the best performance) were compared to ECG ($H\!R_{R}$) using Wilcoxon pair test. The statistically significant difference in medians of pair data was not shown in 24 cases of 27 (88.9%) for $H\!R_{S1}$ and in 20 cases of 27 (74.1%) for $H\!R_{S2}$. The results of pair test (*p*-values) for the individual subjects are presented in Table 6.

**Table 6:** Results of a Wilcoxon signed-rank test of $H\!R$ obtained across subjects from P3; *p*-values lower than the selected significance level (0.05) are marked in bold.

Volunteer #	${H\!R}_{R}-{H\!R}_{S1}$	${H\!R}_{R}-{H\!R}_{S2}$
1	0.704	0.358
2	0.558	**0.050**
3	0.707	**0.035**
4	0.409	0.935
5	0.414	<**0.001**
6	0.577	0.291
7	0.833	0.252
8	**0.014**	0.077
9	0.670	0.660
10	0.758	0.056
11	0.837	0.606
12	0.759	0.739
13	0.498	0.153
14	0.064	0.449
15	0.912	0.957
16	0.431	0.644
17	**0.003**	0.139
18	0.759	0.345
19	0.402	0.164
20	0.784	0.864
21	0.913	<**0.001**
22	0.721	<**0.001**
23	**0.003**	<**0.001**
24	0.179	0.675
25	0.067	**0.001**
26	0.087	0.939
27	0.407	0.864

## Discussion

In this study, the designed low-cost and easy-to-use sensor for PCG measurement was successfully tested. Thanks to the use of non-metallic materials, the sensor is suitable for MRI environments, for which it was primarily designed. The main goal of the study was to determine where on the patient’s chest the designed sensor should be placed to obtain a high-quality signal. While this study does not provide a general standardization of sensor placement for PCG measurement, it identifies the appropriate position for placing the sensors designed in this study. The best signal quality was achieved from position P3 with a median of sensitivity 98.5% for S1 and 97.5% for S2. This position can be chosen for measurements using the designed sensors and also serves as a recommended position for future studies.

The feasibility of using these sensors for HR measurement was demonstrated by comparing the HR data obtained from the R–R, S1–S1, and S2–S2 intervals. Wilcoxon’s signed-rank test showed that the majority of results are comparable. Cases where the p-value was lower than the selected significance level could arise due to improper sensor attachment, ambient noise, or signal pathologies. Generally, the used system (see Fig. 3) composed from microphone *GRAS 40PP-10*, PVC tube, and 3D-printed sensor provides a frequency response up to 100 Hz suitable for detection of S1 and S2. However, according to the literature^12^, S2 is composed also with frequencies higher than 100 Hz, whose response could provide higher quality of S2 detection in both healthy and unhealthy patients.

In Table 7, the results of the proposed study are compared with the recent studies (from 2017 onwards) that focus on a similar topic (i.e., detection of heart sounds) or provide detailed descriptions of sensor placement. These studies are compared based on used dataset, processing methodology, sensor placement (if specified), and evaluation parameters. The former studies are described and compared in the comprehensive review by Ismail et al.^10^.

Because the mentioned studies use existing databases (PhysioNet^58^, PASCAL^59^, eGeneralMedical^60^, Michigan heart sound and murmur database^61^) to test their algorithms, they do not focus on the sensor placement itself. It is important to note that the PhysioNet database only describes common auscultation areas, including the aortic area, pulmonic area, tricuspid area, and mitral area. Additionally, in our case, we identified heart sounds based on the R and T wave identification method^23^. Other studies, however, rely on the algorithm^62^, which uses the R peak and the end of the T wave, or employ different methods for sound localization. Furthermore, the evaluation criteria for correct detecting S1 and S2 can vary among studies, with different techniques and metrics being used. In addition to sensitivity (SE) and positive predictive value (PPV), authors may also employ metrics such as accuracy (ACC), specificity (SP), or precision for evaluation.

**Table 7:** Comparison of results with the latest studies focused on PCG signal processing.

Study	Preprocessing	Dataset	Sensor placement	Evaluation metrics
Nogueira et al.^63^	Mel-frequency cepstral coefficients	PhysioNet challenge 2016 (dataset A)	–	SE (96.47%), SP (72.65%), Overall score (84.56%)
Zhang et al.^64^	Resampling, filtering, and normalization	PASCAL CHSC 2011 (dataset A and dataset B)	–	SE (89–100%), SP (39–90%), Precision (60–76%)
Zhang et al.^65^	Resampling, filtering, and normalization	PASCAL CHSC 2011 and PhysioNet challenge 2016	–	SE (44–100%), SP (39–64%), Precision (25–67%)
Jain et al.^25^	Adaptive method for DWT coefficient reduction for denoising PCG	eGeneralMedical	–	55.1–100% detection of S1 and S2
Fontecave-Jallon et al.^7^	Shannon energy envelope	Private dataset	12 cardiac microphones starting from the top right of the heart and while moving right to left and top to down	Correlation coefficient between S1–S1 and R–R greater than 0.8 for all PCG measurements
Zannat et al.^66^	Shannon energy envelope	Private dataset and PhysioNet challenge 2016	–	–
Prasad et al.^67^	Zero Frequency Filter	PASCAL CHSC 2011 and PhysioNet challenge 2016	–	SE (96.8–98.9%), PPV (99.3–99.4%)
Ozcan et al.^68^	Butterworth band pass filter, Normalization, mel frequency cepstrum with wavelet transform	PASCAL CHSC 2011	–	SE (99,51%), Precision (97,28%)
Ghosh et al.^69^	Stockwell Transform	Michigan heart sound and murmur database	Different auscultation areas such as apex, aortic and pulmonary	ACC 97.5%
Ghosh et al.^70^	Time-Frequency-Domain Deep Neural Network	Michigan heart sound and murmur database and PhysioNet Challenge 2016	–	ACC (95.43–99.55%), SE (97.92–99.93%), SP (98.32–99.26%), Precision (97.60–99.26%)
Proposed	WT and Shannon energy computation (heart sounds classification using K-means)	27 volunteers aged from 21 to 29 years (10 women and 17 men)	7 different locations (the best one was in the middle of sternum)	S1: SE$=$ 98.5%, S1: PPV$=$ 98.0%, S2: SE$=$ 97.5%, S2: PPV $=$ 98.7%

One of the suitable studies for comparing heart rate (HR) could be study^7^, where the authors use a correlation coefficient for comparing $HR_{S1}$ and $HR_R$. In conclusion, they also mention that “determining the optimal localization of the PCG sensor is very difficult, and evaluation depends on many parameters”.

In this study, DWT was used for PCG signal preprocessing since it is a suitable and very popular tool in the area of non-stationary biological signals. Although many studies endeavour to find an optimal parameter setting for PCG denoising^12, 15, 19–21^, it is still not clearly defined and types of maternal wavelets differ across studies. In this paper, the results of wavelet *db12* are presented as the best achieved from previous testing of wavelets *db2–db14* using *Advanced Signal Processing Toolkit* (NI LabVIEW). In future research, other types of maternal wavelets could be tested, e.g. using software platforms that enable a wider range of wavelets for signal processing (e.g., MATLAB). Further research could also explore beyond the discussed DWT, as it is just one of many methods for processing PCG signals. In addition to the mentioned linear filters, PCG signals can be processed using other methods such as Homomorphic filtering^2, 42^, Fast Fourier Transform (FFT), STFT^23, 24, 71, 72^, or Empirical Mode Decomposition (EMD)^73, 74^.

**Figure 14:**
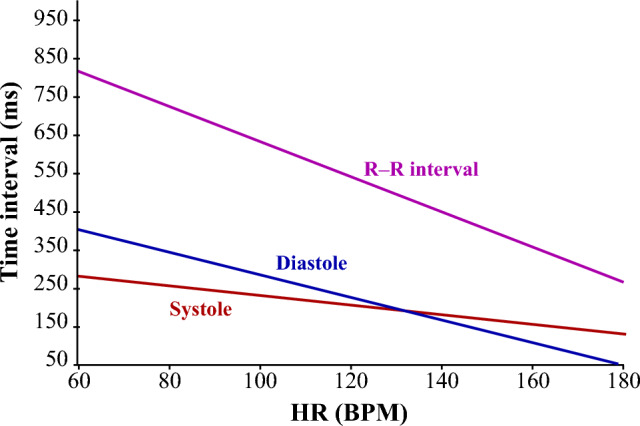
R-R interval, systole and diastole duration depending on heart rate^23^.

One shortcoming of the proposed method could lie in the differentiation of S1 and S2 based on duration of the intervals between heart sounds (using K-means). This approach is suitable for measuring in rest conditions, when ${H\!R}$ is ranged from 60 to 90 bpm. However, during non-resting activities and ${H\!R}$ around 130 bpm, duration of systole and diastole is almost the same, see Fig. 14^23^. In this case, heart sounds should be defined according to another criteria, such as amplitude or time of occurrence after R peak using indirect segmentation. Also, when measuring in motion, a closer contact of the sensor with the patient’s skin and more sophisticated signal processing would be needed, since the signal would be affected by many motion artifacts.

**Figure 15:**
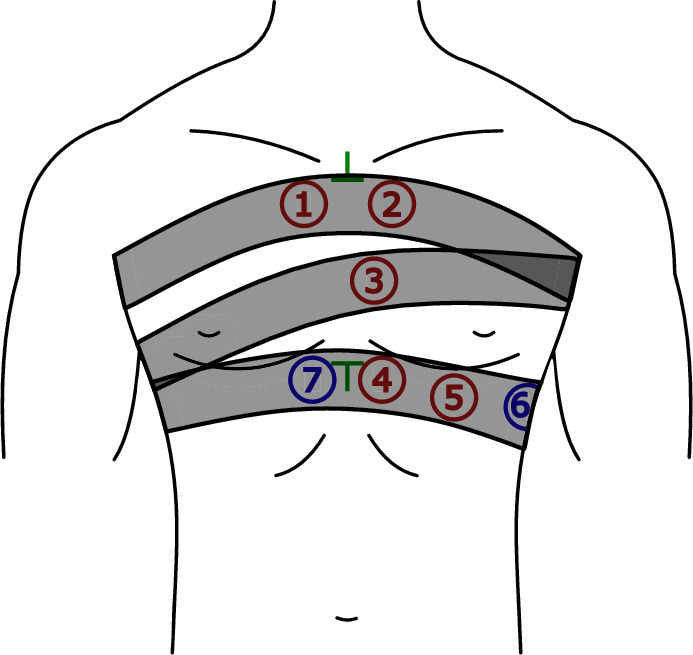
Drawing of three rubber bands fixing the sensors (P1–P7).

The motion artifacts occur also due to breathing and can cause a false HS detection in the proposed approach. The sensors were attached to the body via three rubber strips, as shown in Fig. 15. The proper contact of the sensor with the skin is one of primary preconditions for measuring high-quality PCG signal. However, the attachment of many sensors by one strip was very difficult due to different chest size and shape of the individual subjects. Common fastening of more sensors (P4–P7) can cause insufficient sensor-skin contact and movement during breathing. In these cases, another way of the sensor attachment should be chosen to fix this issue. This observation can explain the best performance of the location P3, where the sensor was fastened by the extra belt.

Future research should involve expanding the selected subjects group. This study focused on a relatively small sample, consisting of 27 healthy individuals aged 21–29 years. Within the study, the appropriate sensor placement and usability were verified among the healthy individuals. To further strengthen the study, future research should explore the dependency of HS detection quality on the patients’ age. Additionally, the proposed approach should be tested in the context of measuring patients with cardiac conditions, as the effects of heart diseases can significantly affect HS detection quality by reducing the amplitude of the S2 sound (as observed in subject #22). Furthermore, the presence of ambient noise in the signal should be considered. Therefore, further studies should verify the usability of the sensors and the proposed algorithm in noisy environments. The designed algorithm should be optimized, such as by adjusting the thresholds $Th_1$ and $Th_2$, or implementing an adaptive threshold that can dynamically adapt its level based on the amplitudes of the S1 and S2 sounds. For the evaluation of pathological or noisy signals, a commercially available PCG device or an evaluation performed by an expert, serving as a reference standard, could be valuable.

### Supplementary Information

Supplementary Information.

## References

[1] Webster JG (2006). Encyclopedia of Medical Devices and Instrumentation.

[2] Ahlström, C. *Nonlinear Phonocardiographic Signal Processing*. Ph.D. thesis, schoolInstitutionen för medicinsk teknik (2008).

[3] Khandpur RS (2020). Compendium of Biomedical Instrumentation.

[4] Ali MN, El-Dahshan E-SA, Yahia AH (2017). Denoising of heart sound signals using discrete wavelet transform. Circuits Syst. Signal Process..

[5] Giordano N, Knaflitz M (2019). A novel method for measuring the timing of heart sound components through digital phonocardiography. Sensors.

[6] Giordano, N. & Knaflitz, M. A method for the estimation of the timing of heart sound components through blind source separation in multi-source phonocardiography. In *2020 IEEE International Symposium on Medical Measurements and Applications (MeMeA)*, 1–6. 10.1109/MeMeA49120.2020.9137315 (IEEE, 2020).

[7] Fontecave-Jallon, J., Fojtik, K. & Rivet, B. Is there an optimal localization of cardio-microphone sensors for phonocardiogram analysis? In *2019 41st Annual International Conference of the IEEE Engineering in Medicine and Biology Society (EMBC)*, 3249–3252. 10.1109/EMBC.2019.8857681 (IEEE, 2019). 10.1109/EMBC.2019.8857681 31946578

[8] Martinek R (2019). A low-cost system for seismocardiography-based cardiac triggering: A practical solution for cardiovascular magnetic resonance imaging at 3 tesla. IEEE Access.

[9] Becker M (2010). Comparison of left ventricular function assessment using phonocardiogram- and electrocardiogram-triggered 2D SSFP CINE MR imaging at 1.5 t and 3.0 t. Eur. Radiol..

[10] Ismail S, Siddiqi I, Akram U (2018). Localization and classification of heart beats in phonocardiography signals—A comprehensive review. EURASIP J. Adv. Signal Process..

[11] Lehner RJ, Rangayyan RM (1987). A three-channel microcomputer system for segmentation and characterization of the phonocardiogram. IEEE Trans. Biomed. Eng..

[12] Nabih-Ali M, El-Dahshan E-SA, Yahia AS (2017). A review of intelligent systems for heart sound signal analysis. J. Med. Eng. Technol..

[13] Meziani F, Debbal SM, Atbi A (2012). Analysis of phonocardiogram signals using wavelet transform. J. Med. Eng. Technol..

[14] Huiying, L., Sakari, L. & Iiro, H. A heart sound segmentation algorithm using wavelet decomposition and reconstruction. In *Proceedings of the 19th Annual International Conference of the IEEE Engineering in Medicine and Biology Society. ’Magnificent Milestones and Emerging Opportunities in Medical Engineering’ (Cat. No.97CH36136)*, 1630–1633. 10.1109/IEMBS.1997.757028 (IEEE, 1997).

[15] Messer SR, Agzarian J, Abbott D (2001). Optimal wavelet denoising for phonocardiograms. Microelectron. J..

[16] Liang, H., Lukkarinen, S. & Hartimo, I. Heart sound segmentation algorithm based on heart sound envelogram. *In Computers in Cardiology* vol. 105–108, (1997). 10.1109/CIC.1997.647841 (IEEE, 1997).

[17] Gupta CN, Palaniappan R, Swaminathan S, Krishnan SM (2007). Neural network classification of homomorphic segmented heart sounds. Appl. Soft Comput..

[18] McGee, S. Chapter 39—Auscultation of the heart: General principles. In McGee, S. (ed.) *Evidence-Based Physical Diagnosis*, 4 edn, 327–332.e1 (Elsevier, Philadelphia, 2018). 10.1016/B978-0-323-39276-1.00039-1.

[19] Chowdhury, M. T. H., Poudel, K. N. & Hu, Y. Automatic phonocardiography analysis using discrete wavelet transform. In *Proceedings of the 3rd International Conference on Vision, Image and Signal Processing*. 10.1145/3387168.3387172 (ACM, 2019).

[20] Marques, N., Almeida, R., Rocha, A. P. & Coimbra, M. Exploring the Stationary Wavelet Transform detail coefficients for detection and identification of the S1 and S2 heart sounds. In *Computing in Cardiology 2013*, vol. 40, 891–894 (2013).

[21] Babaei S, Geranmayeh A (2009). Heart sound reproduction based on neural network classification of cardiac valve disorders using wavelet transforms of PCG signals. Comput. Biol. Med..

[22] Varghees VN, Ramachandran K (2014). A novel heart sound activity detection framework for automated heart sound analysis. Biomed. Signal Process. Control.

[23] El-Segaier M (2005). Computer-based detection and analysis of heart sound and murmur. Ann. Biomed. Eng..

[24] Vikhe, P. S., Nehe, N. S. & Thool, V. R. Heart sound abnormality detection using short time fourier transform and continuous wavelet transform. In *2009 Second International Conference on Emerging Trends in Engineering & Technology*, 50–54. 10.1109/ICETET.2009.112 (IEEE, 2009).

[25] Jain PK, Tiwari AK (2017). An adaptive thresholding method for the wavelet based denoising of phonocardiogram signal. Biomed. Signal Process. Control.

[26] Jain, P. K. & Tiwari, A. K. An adaptive method for shrinking of wavelet coefficients for phonocardiogram denoising. In *2016 IEEE International Conference on Digital Signal Processing (DSP)*. 10.1109/icdsp.2016.7868503 (IEEE, 2016).

[27] Pedrosa, J., Castro, A. & Vinhoza, T. T. V. Automatic heart sound segmentation and murmur detection in pediatric phonocardiograms. In *2014 36th Annual International Conference of the IEEE Engineering in Medicine and Biology Society*. 10.1109/embc.2014.6944078 (IEEE, 2014). 10.1109/EMBC.2014.6944078 25570446

[28] Yiqi Deng, P. J. B. *A robust heart sound segmentation and classification algorithm using wavelet decomposition and spectrogram*. In *Workshop Classifying Heart Sounds, La Palma, Canary Islands* (2012).

[29] Song, D., Jia, L., Lu, Y. & Tao, L. Heart sounds monitor and analysis in noisy environments. In *2012 International Conference on Systems and Informatics (ICSAI2012)*. 10.1109/icsai.2012.6223364 (IEEE, 2012).

[30] Kouras, N., Boutana, D. & Benidir, M. Wavelet based segmentation and time-frequency caracterisation of some abnormal heart sound signals. In *2012 24th International Conference on Microelectronics (ICM)*. 10.1109/icm.2012.6471392 (IEEE, 2012).

[31] Tu, Z., Cao, G., Li, Q., Zhang, X. & Shi, J. Improved methods for detecting main components of heart sounds. In *2010 Sixth International Conference on Natural Computation*. 10.1109/icnc.2010.5584140 (IEEE, 2010).

[32] Quiceno, A., Delgado, E., Vallverd, M., Matijasevic, A. & Castellanos-Domnguez, G. Effective phonocardiogram segmentation using nonlinear dynamic analysis and high-frequency decomposition. In *2008 Computers in Cardiology*. 10.1109/cic.2008.4749002 (IEEE, 2008).

[33] Kumar, D. *et al.* Wavelet transform and simplicity based heart murmur segmentation. In *2006 Computers in Cardiology*, vol. 33, 173–176 (2006).

[34] Wang, P., Kim, Y., Ling, L. & Soh, C. First heart sound detection for phonocardiogram segmentation. In *2005 IEEE Engineering in Medicine and Biology 27th Annual Conference*. 10.1109/iembs.2005.1615733 (IEEE, 2005). 10.1109/IEMBS.2005.1615733 17281503

[35] Liang, H. & Hartimo, I. A heart sound feature extraction algorithm based on wavelet decomposition and reconstruction. In *Proceedings of the 20th Annual International Conference of the IEEE Engineering in Medicine and Biology Society. Vol. 20 Biomedical Engineering Towards the Year 2000 and Beyond (Cat. No.98CH36286)*. 10.1109/iembs.1998.747181 (IEEE, 1998).

[36] Saini M (2016). Proposed algorithm for implementation of Shannon energy envelope for heart sound analysis. Int. J. Electron. Commun. Technol..

[37] Emmanuel BS (2012). A review of signal processing techniques for heart sound analysis in clinical diagnosis. J. Med. Eng. Technol..

[38] PXIe-1092 Specifications. Accessed 31 Aug 2023. https://www.ni.com/docs/en-US/bundle/pxie-1092-specs/page/specs.html.

[39] NI PXIe-7862 specifications. Accessed 31 Aug 2023. https://www.ni.com/docs/en-US/bundle/pxie-7862-specs/page/specs.html.

[40] SCB-68A user manual. Accessed 31 Aug 2023. https://www.ni.com/docs/en-US/bundle/scb-68a-seri/resource/375865a.pdf

[41] GRAS 40PP-10 CCP free-field QC microphone.Accessed 31 Aug 2023. https://www.grasacoustics.com/products/production-line-testing/traditional-production-linemicrophones/product/833-gras-40pp-10-ccp-free-field-qc-microphone.

[42] Gill, D., Gavrieli, N. & Intrator, N. Detection and identification of heart sounds using homomorphic envelogram and self-organizing probabilistic model. In *Computers in Cardiology, 2005*. 10.1109/cic.2005.1588267 (IEEE, 2005).

[43] Wu, C.-H., Lo, C.-W. & Wang, J.-F. Computer-aided analysis and classification of heart sounds based on neural networks and time analysis. In *1995 International Conference on Acoustics, Speech, and Signal Processing*, 3455–3458. 10.1109/ICASSP.1995.479729 (IEEE, 1995).

[44] Gamero, L. & Watrous, R. Detection of the first and second heart sound using probabilistic models. In *Proceedings of the 25th Annual International Conference of the IEEE Engineering in Medicine and Biology Society (IEEE Cat. No.03CH37439)*, 2877–2880. 10.1109/IEMBS.2003.1280519 (IEEE, 2003).

[45] Schmidt SE, Holst-Hansen C, Graff C, Toft E, Struijk JJ (2010). Segmentation of heart sound recordings by a duration-dependent hidden Markov model. Physiol. Meas..

[46] Djebbari, A. & Reguig, F. B. Short-time fourier transform analysis of the phonocardiogram signal. In *ICECS 2000. 7th IEEE International Conference on Electronics, Circuits and Systems (Cat. No.00EX445)*, 844–847. 10.1109/ICECS.2000.913008 (IEEE, 2000).

[47] Sepehri AA, Gharehbaghi A, Dutoit T, Kocharian A, Kiani A (2010). A novel method for pediatric heart sound segmentation without using the ECG. Comput. Methods Programs Biomed..

[48] Choi S, Jiang Z (2008). Comparison of envelope extraction algorithms for cardiac sound signal segmentation. Expert Syst. Appl..

[49] Bravo-Zanoguera, M. E., Medrano, Z. Y., Reyna-Carranza, M. A., Lopez-Avitia, R. & Arriola, H. Simultaneous capture and display of electrocardiogram and multi-site phonocardiogram. In *2009 Pan American Health Care Exchanges*, 26–28. 10.1109/PAHCE.2009.5158358 (IEEE, Mexico City, Mexico, 2009).

[50] Cozic M, Durand LG, Guardo R (1998). Development of a cardiac acoustic mapping system. Med. Biol. Eng. Comput..

[51] Baykal A, Ziya Ider Y, Koymen H (1995). Distribution of aortic mechanical prosthetic valve closure sound model parameters on the surface of the chest. IEEE Trans. Biomed. Eng..

[52] Kolb F, Spanke J, Winkelmann A (2018). Auf den spuren des erb’schen auskultationspunkts. Rätsel gelöst. DMW - Deutsche Medizinische Wochenschrift.

[53] Cheng, L., Carlson, E., Vairavan, S. & Xu, M. Fetal heart rate extraction from maternal abdominal ECG recordings. US Patent 10,531,801. 2020. https://patents.google.com/patent/US10531801B2/en.

[54] Li C, Zheng C, Tai C (1995). Detection of ECG characteristic points using wavelet transforms. IEEE Trans. Biomed. Eng..

[55] Ghaffari A, Golbayani H, Ghasemi M (2008). A new mathematical based QRS detector using continuous wavelet transform. Comput. Electr. Eng..

[56] Du P, Kibbe WA, Lin SM (2006). Improved peak detection in mass spectrum by incorporating continuous wavelet transform-based pattern matching. Bioinformatics.

[57] Litschmannová, M. *Úvod Do Statistiky* (VŠB – TU Ostrava, Fakulta elektrotechniky a informatiky, Ostrava, 2011).

[58] Clifford, G. D. *et al.* Classification of normal/abnormal heart sound recordings: The physionet/computing in cardiology challenge 2016 (2016).

[59] Bentley, P., Nordehn, G., Coimbra, M. & Mannor, S. The PASCAL Classifying Heart Sounds Challenge 2011 (CHSC2011) Results.

[60] Cardiac Auscultation of Heart Murmurs database. http://www.egeneralmedical.com/litohearmur.html.

[61] Judge, R. & Mangrulkar, R. Michigan heart sound and murmur database (mhsdb). https://www.med.umich.edu/lrc/psb_open/html/repo/primer_heartsound/primer_heartsound.html.

[62] Springer D, Tarassenko L, Clifford G (2015). Logistic regression-HSMM-based heart sound segmentation. IEEE Trans. Biomed. Eng..

[63] Nogueira DM, Ferreira CA, Gomes EF, Jorge AM (2019). Classifying heart sounds using images of motifs, MFCC and temporal features. J. Med. Syst..

[64] Zhang W, Han J, Deng S (2017). Heart sound classification based on scaled spectrogram and tensor decomposition. Expert Syst. Appl..

[65] Zhang W, Han J, Deng S (2017). Heart sound classification based on scaled spectrogram and partial least squares regression. Biomed. Signal Process. Control.

[66] Zannat, F., Khan, M. M. & Sohad, S. A. Automated system for features extraction from pcg signal. In *2021 5th International Conference on Computing Methodologies and Communication (ICCMC)*, 1–7. 10.1109/ICCMC51019.2021.9418229 (IEEE, 2021-4-8).

[67] Prasad, R., Yilmaz, G., Chetelat, O. & Magimai.-Doss, M. Detection of s1 and s2 locations in phonocardiogram signals using zero frequency filter. In *ICASSP 2020 - 2020 IEEE International Conference on Acoustics, Speech and Signal Processing (ICASSP)*, 1254–1258. 10.1109/ICASSP40776.2020.9053155 (IEEE, 2020).

[68] Ozkan, I., Yilmaz, A. & Celebi, G. Improved segmentation with dynamic threshold adjustment for phonocardiography recordings. In *2019 41st Annual International Conference of the IEEE Engineering in Medicine and Biology Society (EMBC)*, 6681–6684. 10.1109/EMBC.2019.8856714 (IEEE, 2019). 10.1109/EMBC.2019.8856714 31947374

[69] Ghosh, S. K. & Ponnalagu, R. N. A novel algorithm based on Stockwell transform for boundary detection and segmentation of heart sound components from PCG signal. In *2019 IEEE 16th India Council International Conference (INDICON)*, 1–4. 10.1109/INDICON47234.2019.9030299 (IEEE, 2019).

[70] Ghosh SK, Ponnalagu RN, Tripathy RK, Panda G, Pachori RB (2022). Automated heart sound activity detection from PCG signal using time-frequency-domain deep neural network. IEEE Trans. Instrum. Meas..

[71] Leng S (2015). The electronic stethoscope. Biomed. Eng. Online.

[72] Djebbari, A. & Bereksi Reguig, F. Short-time Fourier transform analysis of the phonocardiogram signal. In *ICECS 2000. 7th IEEE International Conference on Electronics, Circuits and Systems (Cat. No.00EX445)*, vol. 2, 844–847. 10.1109/ICECS.2000.913008 (IEEE, Jounieh, Lebanon, 2000).

[73] Aziz S, Khan MU, Alhaisoni M, Akram T, Altaf M (2020). Phonocardiogram signal processing for automatic diagnosis of congenital heart disorders through fusion of temporal and cepstral features. Sensors.

[74] Chakrabarti, T., Saha, S., Roy, S. & Chel, I. Phonocardiogram signal analysis - practices, trends and challenges. A critical review. In *2015 International Conference and Workshop on Computing and Communication (IEMCON)*, 1–4. 10.1109/IEMCON.2015.7344426 (IEEE, 2015).

